# Verification method of Monte Carlo codes for transport processes with arbitrary accuracy

**DOI:** 10.1038/s41598-021-98429-3

**Published:** 2021-09-30

**Authors:** Fabrizio Martelli, Federico Tommasi, Angelo Sassaroli, Lorenzo Fini, Stefano Cavalieri

**Affiliations:** 1 Dipartimento di Fisica e Astronomia dell’Università degli Studi di Firenze, via Giovanni Sansone 1, 50019 Sesto Fiorentino, Italy; 2 Department of Biomedical Engineering, Tufts University, 4 Colby Street, Medford, MA 02155 USA

**Keywords:** Mathematics and computing, Optics and photonics, Physics

## Abstract

In this work, we present a robust and powerful method for the verification, with arbitrary accuracy, of Monte Carlo codes for simulating random walks in complex media. Such random walks are typical of photon propagation in turbid media, scattering of particles, i.e., neutrons in a nuclear reactor or animal/humans’ migration. Among the numerous applications, Monte Carlo method is also considered a gold standard for numerically “solving” the scalar radiative transport equation even in complex geometries and distributions of the optical properties. In this work, we apply the verification method to a Monte Carlo code which is a forward problem solver extensively used for typical applications in the field of tissue optics. The method is based on the well-known law of average path length invariance when the entrance of the entities/particles in a medium obeys to a simple cosine law, i.e., Lambertian entrance, and annihilation of particles inside the medium is absent. By using this law we achieve two important points: (1) the invariance of the average path length guarantees that the expected value is known regardless of the complexity of the medium; (2) the accuracy of a Monte Carlo code can be assessed by simple statistical tests. We will show that we can reach an arbitrary accuracy of the estimated average pathlength as the number of simulated trajectories increases. The method can be applied in complete generality versus the scattering and geometrical properties of the medium, as well as in presence of refractive index mismatches in the optical case. In particular, this verification method is reliable to detect inaccuracies in the treatment of boundaries of finite media. The results presented in this paper, obtained by a standard computer machine, show a verification of our Monte Carlo code up to the sixth decimal digit. We discuss how this method can provide a fundamental tool for the verification of Monte Carlo codes in the geometry of interest, without resorting to simpler geometries and uniform distribution of the scattering properties.

## Introduction

Monte Carlo (MC) methods for transport processes have been crucial for calculating physical measurands used in the description of complex phenomena. These processes encompass very different fields, involving different particles or different kinds of entities that undergo random walks during their propagation through a medium. In physics, transport processes are fundamental, for instance, in tissue optics^1–10^ atmospheric physics^11,12^, neutron scattering^13,14^ random laser^15–17^, lévy glass^18^, photovoltaic^19–22^, and sensing^23–25^. In biology, they include random walks of animals^26^ and epidemic spreads^27^, whereas in sociology, the random walk emerges in phenomena such as the human migration^28^. In photon migration through turbid media, the radiative transport treatment must include the presence of refraction and reflection effects. A fundamental point for all MC simulations, whatever be the nature of the particles/entities involved, is the availability of a verification method to assure results free of systematic errors and with potentially arbitrary accuracy. This verification is necessary before the results of MC simulations can be considered a gold standard in transport phenomena^2,3,29^. More in general, a MC code (as any other code that “solves” integro-differential equations) should go through verification and validation procedures^30,31^. By the term “verification” is meant a series of procedures to ascertain that a code is computationally sound, in other words that it carries out the calculations it is supposed to, without any bugs^30,31^. While for “validation” is meant the comparison between the numerical results generated by a code with experimental results from a physical system that the code is supposed to model^30,32^. In this work, we propose a verification method for MC codes devised for “solving” the transport equation. We focus on the applications in the field of tissue optics. We do expect that the verification method here proposed can also be applied to MC codes in other fields where the propagation of a random walker can be described by the transport equation.

In principle, a verification method of a forward solver MC code should at first guarantee that the trajectories generated by the code are statistically correct, i.e., consistent with the statistical laws of radiative transport. Further, the trajectories must take into account a correct treatment of boundaries. In general, the existing literature^1,3,33–42^ shows three different procedures typically used in biomedical optics to verify MC codes, based on comparisons with: (1) previously verified MC codes^33–37,39–42^; (2) solutions of the radiative transfer equation (RTE) (in tissue optics have been largely used the reference RTE results provided by van del Hulst^43^ and Giovanelli^1,34,36,44^); (3) asymptotic solutions of the diffusion equation^35,37,38^. The use of existing verified MC codes is mainly limited by the finite accuracy of the MC data generated. Benchmark solutions of RTE, mostly in semi-analytical form, are only available for regularly bounded geometries, e.g. semi-infinite, slab, layers, and mostly for uniform scattering properties^45–48^. This verification method has two drawbacks: these solutions cannot be usually expressed in closed form and therefore they are known with limited accuracy and they are also likely to be affected by convergence problems of the calculated quantities. Finally, the asymptotic solutions of the diffusion equation used in the literature^35^ are limited by the intrinsic approximations of the diffusion theory and cannot be used in all the cases covered by the RTE. Moreover, for finite geometries and in cases with scattering and/or refractive index mismatch between different regions of the medium the accuracy of the solutions of the diffusion equation is further limited^3^.

In the literature a general reliable method based on a large class of closed form exact RTE solutions usable for the verification of MC codes, is still missing. This work proposes a verification procedure of Monte Carlo codes that is a contribution to fill this gap. The proposed method, compared to previous used semi-analytical benchmarks^30,45–48^, is able to test the correctness of MC codes in more complex geometries and/or distribution of the optical properties. The procedure is capable of detecting various types of errors in a MC code and is particularly sensible to inaccuracies in the treatment of boundaries. Typical ones are related to the wrong model of light propagation (including the interaction with boundaries), or in the implementation of the actual code, including systematic errors due to the finite accuracy of some built-in functions^41,33^. We will show that in a correct MC code the accuracy can be potentially arbitrary and most importantly, consistent with the precision of the calculation. The reference used for the verification is expressed by extremely simple solutions that, being independent of scattering properties and detailed geometric characteristics of the medium, offer a very large range of applications. It is finally worth to note that the reference solutions here proposed for the verification of MC codes have an extremely simple mathematical form that makes their use open to the non-experts of RTE solutions and numerical methods.

The core of the method lies in the well known, but not as much subjected to usability, invariance property (IP) of the mean path length $\langle L \rangle$ spent by a random walker within a disordered medium, once its entrance into the medium takes place homogeneously and isotropically and no particle absorption or annihilation is present in the medium^49–51^. In the optical case, this condition corresponds to a constant incoming radiance, usually denoted as Lambertian illumination. If such entrance condition is assumed, the result for $\langle L \rangle$ leads to, at a first glance, quite counter-intuitive and surprising value: a constant quantity that only depends on the basic geometric characteristics of the medium regardless of the distribution of the scattering properties. In the optical case, these results can be generalized, also taking into account the refractive index mismatch between the external environment and the medium and also the mismatches among different regions of the same medium^52^. Indicating with *V*, *S* and *P* the volume, the surface (for a 3D medium) and the perimeter (for a 2D medium) respectively, the predicted values by the IP, in 2D and 3D domains, assume very simple forms^52^:

1 $$\begin{aligned} \langle L \rangle _{IP}^{3D}=4\frac{V}{S}n_r^2,\qquad \langle L \rangle _{IP}^{2D}=\pi \frac{S}{P}n_r. \end{aligned}$$

The introduction of the relative refractive index $n_r=n/n_e$, where *n* is the refractive index of the medium and $n_e$ of the external medium^52,53^, refers to the optical case ($n_r$ can be simply set as 1 in other contexts). The only restriction of validity of the above property is for the case $n_r>1$ for which it must be imposed, for nonergodic geometries (e.g. sphere and slab), a scattering coefficient $\mu _s>0$^54,55^. Except this special case, the property is valid for any distribution of the scattering coefficient and scattering function inside the medium. Equation (1) is thus also valid in presence of scattering inhomogeneities in the medium^56^ and also for refractive index mismatches between different regions of the propagation domain^52,55^. Also known as “Cauchy’s formula”, the invariance property has been studied in different contexts, such as nuclear physics^56,57^ and optics^22,52,58^, and has been recently experimentally verified for light propagation through scattering media^53^ and for bacterial random walks^19^.

In the optical case, for an inhomogeneous volume *V* that can be divided in a number *N* of discrete sub-volumes $V_k$ of refractive index $n_k$ and with arbitrary scattering properties inside each $V_k$, Eq. (1) can be generalized as follows^52,55^:

2 $$\begin{aligned} \langle L \rangle _{IP}^{}=\sum \limits _{k=1}^{N}\langle L_k \rangle _{IP}=4\frac{\sum \limits _{k=1}^{N}V_k\left( \frac{n_k}{n_e}\right) ^2}{S} , \end{aligned}$$

where *S* is the surface of the whole medium. From Eq.(2) we note that $\langle L_k \rangle _{IP}$ is expressed for the volume $V_k$ as^55^:

3 $$\begin{aligned} \langle L_k \rangle _{IP}=4 \left( \frac{n_k}{n_e}\right) ^2 \frac{V_k}{S}. \end{aligned}$$

The above properties are valid in complete generality except the cases characterized by $n_k>n_e$ for which we must assume, for nonergodic geometries (e.g. sphere and slab), a non null scattering coefficient, $\mu _{sk}$, inside each volume $V_k$^54,55^. It is important to note that for $n_k>n_e$ we have a discontinuity of $\langle L_k \rangle$ between the case $\mu _s=0$ and the case $\mu _s\ne 0$^54,55^. This discontinuity generates the invalidity of the IP for $n_k>n_e$ and $\mu _{sk}=0$. All the above expressions for $\langle L \rangle _{IP}$ and $\langle L_k \rangle _{IP}$ are at the basis of the method proposed in this work. The simple closed form of Eqs. (1)–(3) allows for a comparison with the Monte Carlo results arbitrarily accurate that can be referred to both homogeneous and inhomogeneous media with a convex external boundary. The use of the above exact closed form analytical solutions (Eqs. (1)–(3)) shows that the verification method here proposed is sensible to the refractive index mismatch between two regions of the medium or with the external region. Thus, this fact implies that the method exploits a reference that can reveal inaccuracies in the treatment of boundaries in finite media.

In this work we have used the proposed procedure to demonstrate the validity of our MC code that simulates light propagation through turbid media with refractive index mismatches and values of the scattering coefficient ranging over eight orders of magnitude. The verification is easily carried out by comparing the mean pathlength calculated by the MC code with the theoretical value provided by IP. By doing so, we have verified our code up to six significant decimal digits. Last but not least, we will show how this verification method can detect systematic errors due to the finite precision of numeric computation. The effectiveness of the method has been verified by the application of statistical tests.

## Results

This section is devoted to show the power and the accuracy of the proposed method by applying it to our MC code, taken as example of a typical program for generating random trajectories^3^. Since the code has been used in the field of tissue optics, it also allows to use the IP method with media with refractive index mismatches. All the presented results provide a direct comparison between the calculated values by the MC simulation and the exact theoretical reference of the IP allowing the achievement of arbitrary accuracy only limited by the finite computation time and by the finite precision exploited in the calculations. For the details of the MC code, we refer to the “Methods” section.

The results presented in this section pertain to geometries such as the sphere and the slab. In the supplemental material also results for the cylinder have been reported. It is worth to keep in mind that the simplicity of the considered geometry does not affect the extent of the verification carried out that addresses all the intrinsic computational procedures of the code. In fact, the proposed method of verification can be used for any kind of geometry, once the boundary conditions and the illumination of the medium are correctly implemented. In this work, we show results in a layered sphere and slab (see “Inhomogeneous media” section) for which there are no semi-analytical solutions of RTE.

The results of this section are divided into three main parts: homogeneous media, inhomogeneous media and special tests.

**Figure 1:**
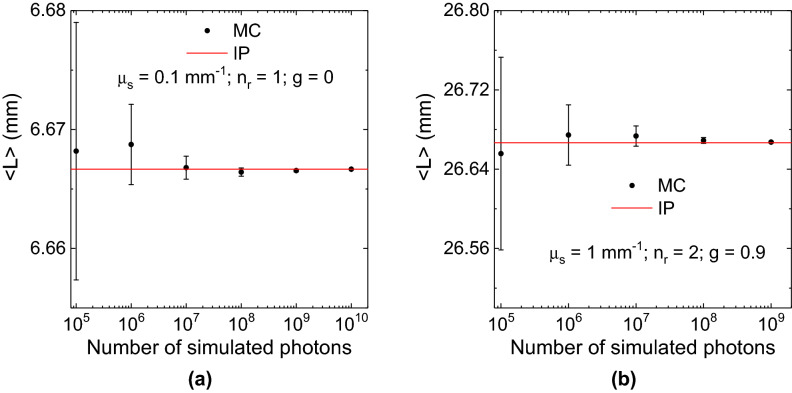
Homogeneous non-absorbing sphere of radius $r=$ 5 mm. Figure (**a**) pertains to a scattering coefficient $\mu _s$ = 0.1 $\hbox {mm}^{-1}$, $n_r=1$ and $g=0$, whilst figure (**b**) pertains to a scattering coefficient $\mu _s$ = 1 $\hbox {mm}^{-1}$, $n_r=2$ and $g=0.9$. The error bar is the standard error, i.e., the standard deviation of $\langle L \rangle$ calculated for different numbers of simulated photons.

**Table 1:** Data for the sphere of radius $r=5$ mm and $n_r=1$ for different values of the scattering coefficient $\mu _s$ and different numbers *N* of generated trajectories.

$\langle L \rangle _{IP}=6.\bar{6}$ mm	N = $10^4$		N = $10^5$		N = $10^6$		N = $10^7$		N = $10^8$		N = $10^9$		N = $10^{10}$	
$\mu _s$ ($\hbox {mm}^{-1}$)	$\langle L \rangle$ (mm)	$\sigma _{\langle L \rangle }$ (mm)	$\langle L \rangle$ (mm)	$\sigma _{\langle L \rangle }$ (mm)	$\langle L \rangle$ (mm)	$\sigma _{\langle L \rangle }$ (mm)	$\langle L \rangle$ (mm)	$\sigma _{\langle L \rangle }$ (mm)	$\langle L \rangle$ (mm)	$\sigma _{\langle L \rangle }$ (mm)	$\langle L \rangle$ (mm)	$\sigma _{\langle L \rangle }$ (mm)	$\langle L \rangle$ (mm)	$\sigma _{\langle L \rangle }$ (mm)
0	6.645	0.025	6.6760	0.0073	6.6650	0.0023	6.66731	0.00082	6.66650	0.00020	6.666658	0.000079	6.666698	0.000028
$10^{-7}$	6.644	0.025	6.6753	0.0067	6.6672	0.0024	6.66743	0.00077	6.66683	0.00024	6.666599	0.000079	6.666676	0.000022
$10^{-6}$	6.616	0.021	6.6667	0.0078	6.6628	0.0022	6.66606	0.00072	6.66683	0.00025	6.666660	0.000070	6.666678	0.000023
$10^{-5}$	6.636	0.021	6.6624	0.0080	6.6690	0.0022	6.66649	0.00077	6.66644	0.00024	6.666544	0.000072	6.666637	0.000022
$10^{-4}$	6.656	0.024	6.6673	0.0072	6.6622	0.0025	6.66607	0.00081	6.66672	0.00023	6.666762	0.000079	6.666677	0.000028
$10^{-3}$	6.678	0.021	6.6727	0.0081	6.6665	0.0024	6.66708	0.00080	6.66666	0.00029	6.666606	0.000071	6.666687	0.000026
$10^{-2}$	6.618	0.026	6.6655	0.0074	6.6699	0.0025	6.66744	0.00076	6.66645	0.00021	6.666737	0.000082	6.666669	0.000024
$10^{-1}$	6.645	0.034	6.668	0.011	6.6687	0.0034	6.6668	0.0010	6.66642	0.00035	6.66655	0.00011	6.666664	0.000033
1	6.714	0.087	6.703	0.028	6.6640	0.0081	6.6612	0.0024	6.66543	0.00092	6.66671	0.00027	6.666559	0.000088
10	6.41	0.38	6.63	0.11	6.697	0.035	6.670	0.013	6.6673	0.0041	6.6656	0.0012	6.66687	0.00036

The value predicted by theory is $\langle L \rangle _{IP}=\frac{4}{3}r=6.\bar{6}$ mm. $\sigma _{\langle L \rangle }$ is the standard deviation of $\langle L \rangle$. In the Supplemental Material a version of this table showing the deviation between $\langle L \rangle _{MC}$ and $\langle L \rangle _{IP}$ is also reported.

**Figure 2:**
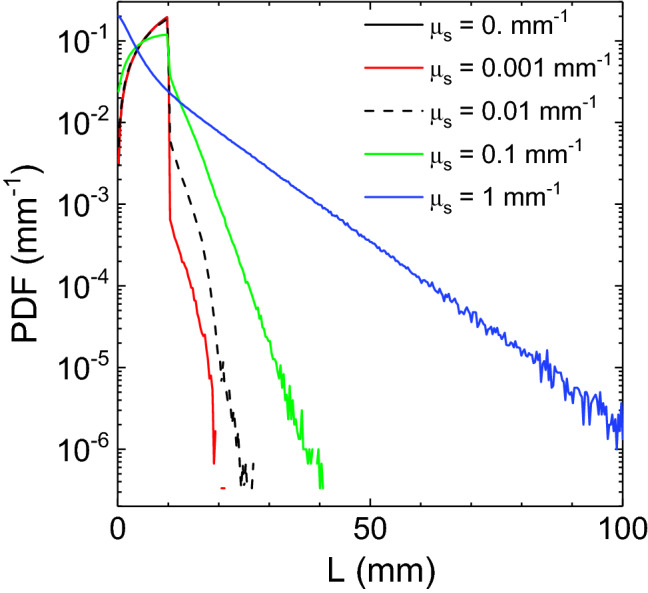
Path length probability distribution function, *PDF*(*L*), versus the length *L* obtained with MC simulations for a sphere of 5 mm radius, $n_r=1$, for different values of $\mu _s$. In figure the case $\mu _s = 0$ is undistinguishable from the case $\mu _s$ = 0.001 $\hbox {mm}^{-1}$ for $L<$10 mm, while being the ballistic component is zero for $L>$10 mm.

**Figure 3:**
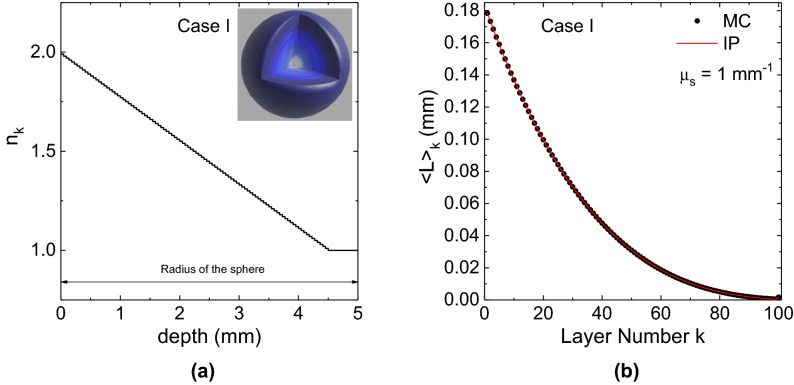
(**a**) Refractive index profile inside the sphere versus the depth from the external surface. (**b**) Partial pathlength in each layer $\langle L_k \rangle$ is shown for a hundred-layered sphere (**a**) with a refractive index that decreases towards the center. The innermost layer is a sphere of radius 0.5 mm. The MC results shown in figure pertain to $N=10^7$ simulated trajectories. Figures created by using OriginPro 2021b Academic (https://www.originlab.com/2021).

**Figure 4:**
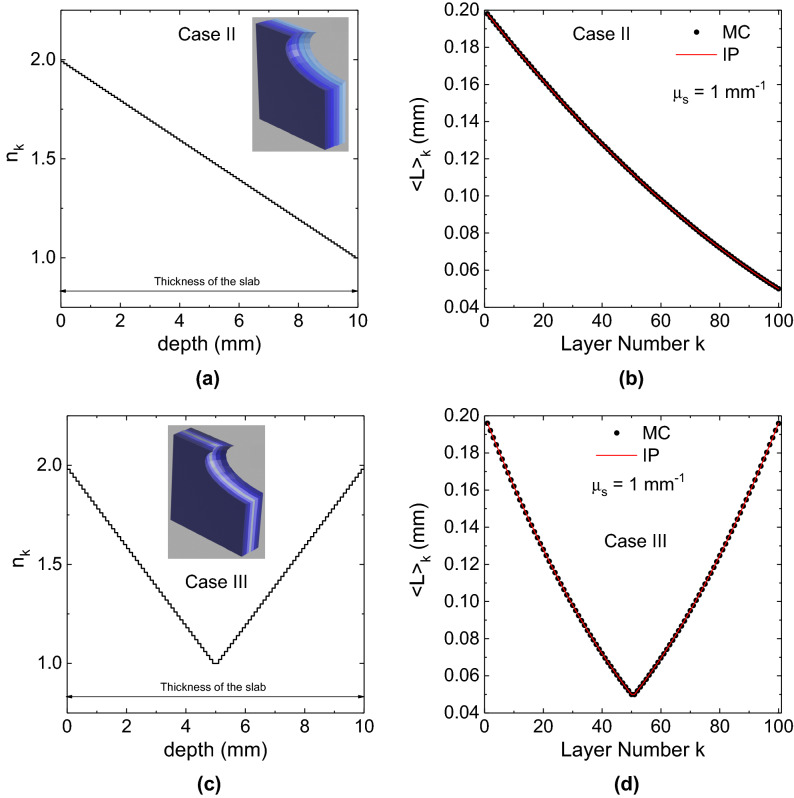
(**a,c**) Refractive index profile inside the slab versus the depth from the external surface ($z=0$) (cases II and III). (**b,d**) Shows the partial pathlength $\langle L_k \rangle$ for a hundred-layered slab for the two distribution of refractive index shown in (**a,c**). The MC results shown in figure pertain to $N=10^7$ simulated trajectories. Figures created by using OriginPro 2021b Academic (https://www.originlab.com/2021).

### Homogeneous media

Figure 1a shows the results of the MC calculation of the average path length spent by photons in an isotropically illuminated, homogeneous, spherical medium of radius $r=$ 5 mm, scattering coefficient $\mu _s$ = 0.1 $\hbox {mm}^{-1}$ and relative refractive index $n_r=1$. The scattering function, based on the Henyey and Greenstein (HG) model^3,59^, has an asymmetric factor $g=0$. According to Eq. (1), in this case the value predicted by theory is $\langle L \rangle _{IP}=\frac{4}{3}r$. In Fig. 1b the case of a sphere with the same radius $r=$ 5 mm, $\mu _s$ = 1 $\hbox {mm}^{-1}$, $g=0.9$ and $n_r$ = 2 is shown. In this case the corresponding IP average pathlength is: $\langle L \rangle _{IP}=\frac{16}{3}r$. In these two examples it is evident the expected convergence of $\langle L \rangle _{MC}$ to the predicted value $\langle L \rangle _{IP}$ when the number of trajectories *N* increases. Moreover, as expected, the standard deviation of the mean $\sigma _{\langle L \rangle }$, is such that $\sigma _{\langle L \rangle }=\sigma _L/\sqrt{N}$, where $\sigma _L$ is the standard deviation of the pathlengths of simulated photons. For instance, for $\mu _s=0.1$ $\hbox {mm}^{-1}$ in Fig. 1a the ratio between $\sigma _{\langle L \rangle }^{ N=10^{4}}$ and $\sigma _{\langle L \rangle }^{N=10^{10}}$ is $\sim 1000$ (see Table 1) which is in agreement with the theoretical scaling of $\sigma _{\langle L \rangle }$. The results of Fig. 1b highlight the robustness of the method with a different scattering phase function. The form of $\sigma _{\langle L \rangle }$ can shed some light on the fact that it increases when the scattering of the medium increases. In fact, $\sigma _{\langle L \rangle }$ is directly related to the form of $\sigma _L$ that can be expressed as $\sigma _L^2=\langle L^2 \rangle - \langle L \rangle ^2$. A direct insight on the behaviour of $\sigma _L$ can be obtained by plotting the path length Probability Distribution Function, PDF(L), versus the scattering coefficient of the medium $\mu _s$. In Fig. 2 the PDF(L) for a sphere 5 mm radius and $n_r=1$ is shown. The PDF(L) is plotted versus $\mu _s$ with different curves characterized by the the same $\langle L \rangle$ equal to $\langle L \rangle _{IP}$. However, they also show larger values of $\langle L^2 \rangle$ for larger scattering coefficients, and this explains the observed behaviour of $\sigma _{\langle L \rangle }$ calculated with the MC simulations (see Table 1).

In Table 1, the data corresponding to $\langle L \rangle$ for the first case of Fig. 1 (HG scattering phase function with $g=$ 0) are reported for different values of *N* and for $\mu _s$ varying in a wide range of values. The results clearly show that for all the situations of the table the value of $\langle L \rangle$ is consistent within one standard deviation with the expected value given by Eq. (1). We have run seventy t-tests for all the cases reported in Table 1, at a 5% level of significance and we found that, only two MC—calculated average path lengths deviated from the IP theoretical value, i.e., the null hypothesis was rejected. However, no correction was applied for this multiple comparison design of the test, and about three values are expected to deviate by mere chance, from the IP theoretical value. It is worth to note that the MC calculated $\langle L \rangle$ is accurate up to six decimal significant digits.

Further cases, for different geometries, scattering and refractive index mismatch, are reported in the supplementary material. For an anisotropic scattering function (HG model with $g=0.9$) similar results to those here shown have been also obtained. For very low scattering, we note a slower convergence, versus the number of simulated trajectories, of the MC results to the IP value when $n_r>1$ if compared to the other cases with $n_r\le 1$. The reason of this behaviour lies in the presence of rare long photons trajectories that when $n_r>1$ can be established inside the volume with a regime of guided propagation. The guided propagation of this kind of trajectories can only be neutralized by one scattering event inside the medium. The effect is present for the geometries of sphere, slab and cylinder^54,55^. The insufficient sampling of this kind of trajectories may lead to an inaccurate evaluation of $\langle L \rangle$. The effect is particularly important for very low values of the scattering coefficient, while it vanishes as the scattering increases.

### Inhomogeneous media

Since the IP can be generalized in an inhomogeneous medium by expressing the average partial internal path length $\langle L_k \rangle$ spent in a sub-volume $V_k$ of a medium of external surface *S* (see “Introduction” section)^55^, the method of verification can be also extended to any portion of the volume.

In general, any sub-volume in an inhomogeneous medium can be characterized by a different $\mu _{s}$, scattering function and $n_k$. This approach provides the possibility to verify the IP, and then the correctness of the code, in any sub-domain of an inhomogeneous medium, as shown in Figs. 3 and 4. In the three examples, any sub-volume consists in a layer of different refractive index for a medium of 100 layers. Three cases are considered: a layered sphere (Fig. 3) and two layered slabs (Fig. 4). In Figs. 3 and 4 the profiles of the refractive index inside the three layered media is shown versus the depth from the external surface. In the pictures appearing in the inset of Figs. 3 and 4 a darker blue colour means a larger refractive index. The external environment has a refractive index $n_e=2$. In the case of the sphere of radius of 5 mm (case I) the refractive index decreases with the depth from $n_1=1.99$ up to $n_{100}=1.00$ ($n_k=n_e-k\times 0.01$). The innermost layer is a sphere of radius 0.5 mm. In the other two cases (II and III), the medium is a layered laterally-infinite slab of thickness of 10 mm with two different refractive index profiles. In the case II (see Fig. 4a), the refractive index decreases from the left side one ($n_1=1.99$) to the opposite one ($n_{100}=1.00$), i.e., $n_k=n_e-k\times 0.01$, whereas in the case III (see Fig. 4b) it decreases from one side of the slab towards the center and it increases again towards the other side. Therefore we have: $n_1=n_{100}=1.98$ and $n_{50}=n_{51}=1.00$). The rules to assign the refractive index to a layer are: $n_k=n_e-k\times 0.02$ for $k=1,\dots , 50$, and $n_k=n_e-(101-k)\times 0.02$ for $k=51,\dots , 100$. Then, Figs. 3 and 4 show the value of $\langle L_k \rangle$ calculated by MC, compared to the theoretical value expressed by Eq. (3). The agreement is excellent, with the MC and IP curves almost indistinguishable, showing that the proposed method is also applicable to inhomogeneous media making use of the reference values of the mean internal path length $\langle L_k \rangle$ provided by the IP.

**Table 2:** Data for a 4-layered non-absorbing sphere of external radius $r=5$ mm and internal radii 4, 3 and 2 mm.

$\mu _{s0}$ ($\hbox {mm}^{-1}$)	$\langle L_1 \rangle _{IP}=2.490833$ mm		$\langle L_2 \rangle _{IP}=1.110000$ mm		$\langle L_3 \rangle _{IP}=0.395833$ mm		$\langle L_4 \rangle _{IP}=0.106667$ mm	
	$\langle L_1 \rangle$ (mm)	$\sigma _{\langle L_1 \rangle }$ (mm)	$\langle L_2 \rangle$ (mm)	$\sigma _{\langle L_2 \rangle }$ (mm)	$\langle L_3 \rangle$ (mm)	$\sigma _{\langle L_3 \rangle }$ (mm)	$\langle L_4 \rangle$ (mm)	$\sigma _{\langle L_4 \rangle }$ (mm)
0	2.49068	0.00066	1.10999	0.00053	0.39576	0.00034	0.10699	0.00019
$10^{-6}$	2.49039	0.00061	1.11017	0.00053	0.39575	0.00033	0.10653	0.00019
$10^{-5}$	2.49045	0.00052	1.10986	0.00051	0.39594	0.00036	0.10673	0.00019
$10^{-4}$	2.49136	0.00068	1.10990	0.00052	0.39556	0.00032	0.10667	0.00018
$10^{-3}$	2.49136	0.00062	1.11052	0.00051	0.39574	0.00033	0.10662	0.00018
$10^{-2}$	2.49064	0.00055	1.11020	0.00056	0.39567	0.00036	0.10687	0.00015
$10^{-1}$	2.49165	0.00078	1.10923	0.00053	0.39590	0.00035	0.10687	0.00017
1	2.4904	0.0011	1.10981	0.00093	0.39681	0.00052	0.10682	0.00027

In the first layer, $r\in (4,5)$ mm, $n_1=1.75$ and $\mu _{s}=\mu _{s0}$; in the second layer, $r\in (3,4)$ mm, $n_1=1.5$ and $\mu _{s}=0$; in the third layer, $r\in (2,3)$ mm, $n_1=1.25$ and $\mu _{s}=\mu _{s0}$; and in the forth layer $r\in (0,2)$ mm, $n_1=1$ and $\mu _{s}=0$. The refractive index of the external medium is 2. The results of MC simulations for several values of $\mu _{s0}$ are shown in table for the partial path length $\langle L_k \rangle$ spent in the layers. The values predicted by IP are also shown. The data in table pertain to $N=10^7$ simulated trajectories. In the supplemental material a version of this table showing the deviation between $\langle L \rangle _{MC}$ and $\langle L \rangle _{IP}$ is also reported.

**Table 3:** The effect of a single-precision (first five rows, in bold) random number generator compared to a double-precision one (last five rows, in italic) in a laterally infinite extended slab of thickness 10 mm ($\langle L \rangle _{IP}$ = 20 mm) for $n_r=1$, and for different values of the scattering coefficient.

$\langle L \rangle _{IP}=20$ mm	N = $10^6$		N = $10^7$		N = $10^8$		N = $10^9$		N = $10^{10}$	
$\mu _s$ ($\hbox {mm}^{-1}$)	$\langle L \rangle$ (mm)	$\frac{\langle L \rangle - \langle L \rangle _{IP}}{\sigma _{\langle L \rangle }}$	$\langle L \rangle$ (mm)	$\frac{\langle L \rangle - \langle L \rangle _{IP}}{\sigma _{\langle L \rangle }}$	$\langle L \rangle$ (mm)	$\frac{\langle L \rangle - \langle L \rangle _{IP}}{\sigma _{\langle L \rangle }}$	$\langle L \rangle$ (mm)	$\frac{\langle L \rangle - \langle L \rangle _{IP}}{\sigma _{\langle L \rangle }}$	$\langle L \rangle$ (mm)	$\frac{\langle L \rangle - \langle L \rangle _{IP}}{\sigma _{\langle L \rangle }}$
0	**19.998**	**− 0.045**	**19.996**	**− 0.41**	**19.9947**	**− 1.58**	**19.9956**	***− 3.70***	**19.99537**	***− 18.9***
$10^{-9}$	**20.029**	**0.84**	**19.993**	**− 0.71**	**19.9931**	***− 2.04***	**19.9965**	***− 2.74***	**19.99501**	***− 13.8***
$10^{-7}$	**20.029**	**0.58**	**19.998**	**− 0.18**	**19.9939**	**− 1.86**	**19.9948**	***− 4.51***	**19.99587**	***− 13.1***
$10^{-5}$	**20.019**	**0.43**	**19.993**	**− 0.48**	**19.9945**	**− 1.47**	**19.9968**	***− 3.08***	**19.99668**	***− 7.94***
$10^{-3}$	**20.018**	**0.77**	**19.993**	**− 0.77**	**20.0004**	**0.16**	**20.00037**	**0.41**	**19.99951**	**− 1.80**
0	*19.977*	*− 0.70*	*19.992*	*− 0.67*	*20.0038*	*0.94*	*20.0004*	*0.30*	*19.99958*	*− 0.98*
$10^{-9}$	*19.988*	*− 0.33*	*19.995*	*− 0.44*	*19.9978*	*− 0.61*	*20.0001*	*0.10*	*20.00056*	*1.24*
$10^{-7}$	*19.977*	*− 0.78*	*19.994*	*− 0.51*	*19.9956*	*− 1.16*	*19.9993*	*− 0.53*	*19.99956*	*− 0.96*
$10^{-5}$	*20.045*	*0.77*	*20.009*	*0.80*	*19.9954*	*− 1.27*	*20.0007*	*0.59*	*19.99993*	*− 0.17*
$10^{-3}$	*20.002*	*0.06*	*19.9982*	*− 0.25*	*20.0015*	*0.53*	*20.00208*	***2.61***	*19.9981*	*− 0.33*

The t statistics $\frac{\langle L \rangle _{MC} - \langle L \rangle _{IP}}{\sigma _{\langle L \rangle }}$ of each calculation are reported. The bolditalic values are the ones that can be rejected with the confidence level of 5%.

Similar results to those shown in the previous figures have been also obtained when the scattering properties in the layers are different. In Table 2 are summarized the MC results for a four-layered sphere with the refractive index varying from the external medium to the center of the sphere with the following values: 2, 1.75, 1.5, 1.25 and 1. The scattering from the external layer to the more internal one assumes the following discrete distribution of values: $\mu _{s0}$, 0, $\mu _{s0}$ and 0, with $\mu _{s0}$ in the range [0,1] $\hbox {mm}^{-1}$ (see Table 2). We ran a set of one-sample t tests (at a level of significance of 5%) to compare the MC calculated $\langle L_k \rangle$ of Table 2 with $\langle L_k \rangle$ predicted by the IP. The Null hypothesis was rejected only one time, which is expected by the multiple comparison nature of the test design. Thus, the method can also be successfully applied by using statistical tests on $\langle L_k \rangle$.

Similar results have been also obtained for a layered sphere of one thousand layers and for layered spheres with different values of the scattering coefficient in each layer. It is worth to note that the presented results cannot be obtained by semi-analytical benchmarks that are not available for such complex layered structures of the medium where the scattering properties may change between the layers. As it is shown in the work of Liemert et al.^48^, the solution of RTE for a three-layer slab is already a formidable problem. We finally note that, the presented results for layered media are of particular interest in the field of tissue optics since many organs have a layered structure.

### Special tests

In addition to the non-statistical errors in the MC code, i.e., model errors, and logical errors in the implementation, the proposed verification method can also detect significant biases caused by the finite precision of the functions used in the calculations. One typical example is related to the sampling of the phase function^60^, and the precision or the periodicity of the pseudo-random number generator^41,33^. Here we show how the choice of a single-precision (*SP*) random number generator, in place of a double-precision (*DP*) one, affects the final results when a very high accuracy is required. The former in exponential representation has mantissa of 7 digits, whereas the latter of 14 digits. To avoid errors due to the sampling of the phase function, we used the Henyey-Greenstein model^59^ which provides an analytical expression for the cumulative probability of a given scattering angle. This is a useful exercise to highlight how the characteristics of the random number generator could restrict the achievable accuracy of the MC results. This example stresses also the importance of the proposed verification method to detect systematic errors hidden inside the code.

In presence of low scattering, given a laterally-infinite 10 mm thick slab as medium with $n_r=1$ (as in the example in Table 3), a critical role for implementing a Lambertian source is played by the entrance angle; indeed, in this case, a limit in extraction of the entrance angle $\theta _{in}$ leads to a lack of very long trajectories and this leads to a non uniform radiance inside the probed volume. Such a non uniformity causes a violation of the hypothesis of equilibrium requested by the IP. Given $\xi$ a uniformly distributed random number in the interval (0, 1), the equation for the Lambertian distributed entrance angle, with respect to the normal to the surface, is $\theta _{in}={\arccos }\left( \sqrt{1-\xi }\right)$. The azimuthal angle $\varphi$ is drawn from a uniform distribution between 0 and $2\pi$. The point is that a SP random number generator yields a smaller maximum incident angle than a DP one. In Table 3, the bias of the MC-calculated $\langle L \rangle$ with respect to the IP value ($\langle L \rangle _{IP}=$ 20 mm) is reported in the case of a SP (first five rows, in bold) and DP (last five rows, in italic) random number generator for $\mu _s\in [0,10^{-3}]$ $\hbox {mm}^{-1}$ and $N=10^6,10^7,10^8,10^9,10^{10}$ trajectories. The effect of the maximum $\theta _{in}$ achievable ($\theta _{in}^{SP}=1.570451\dots \left( 89.98021^\circ \dots \right)$ vs. $\theta _{in}^{DP}=1.570796\dots \left( 89.99998^\circ \dots \right)$) leads to an evident reduction of the bias with the DP. From Table 3, for the case of SP random number generator we can see that an increasing number of t tests are rejected as the number of simulated photons is also increased. The reason is because a large sample of simulated photons is needed to detect a “small” true bias. For the case $\mu _s=0$, the biases can be also theoretically calculated and they are: $b_{SP}\approx -\,0.0069$ mm, $b_{DP}\approx -6.98$ × 10$^{-6}$ mm, for the SP and DP calculations, respectively. Note that for the SP case the number of tests rejected well exceeds what is expected by the multiple comparison design of the test. On the contrary only one value of the biases calculated with the DP random number generator was found significant. To better emphasize the significance of this test, for $N=10^9$ we have also run a simulation where the DP random number is only used to extract the entrance angle $\theta _{in}$. The results obtained are fully consistent with the values for DP random number generator shown in Table 3. This fact implies that the SP random number generator significantly affects only the extraction of $\theta _{in}$ and not the other computational parts of the simulated trajectories. We also note that the results of Table 3 for the case $\mu _s=0$ and SP calculations, are in agreement with theoretical estimation of the bias ($\langle L \rangle -\langle L \rangle _{IP}$) and the variance $\sigma ^2(L)$, where the latter is calculated by neglecting the “forbidden” entrance angles. For this case the “t” statistics becomes significant when $N=10^{8}$ ($t=-\,2.$, $p<0.025$; one-sample one-tailed t test), which is the same order of magnitude of the “t” value of Table 3. If we apply the same theoretical consideration for DP calculations, we estimate that the bias would be “detected” when $N=10^{15}$ ($t=-\,4.35$, $p<0.0005$). This example shows how the proposed method can detect possible systematic errors that manifest in inconsistencies between the bias and the precision of the calculation by simply increasing the number of simulated photons.

## Discussion

In this work we have proposed a general method for testing a MC code for transport phenomena. The results presented show that, in the condition of uniform and isotropic illumination of a medium (Lambertian illumination), the value of $\langle L \rangle$ calculated with an error-free MC method ($\langle L \rangle _{MC}$) converges to the value predicted by the IP ($\langle L \rangle _{IP}$), with an error $\sigma _{\langle L \rangle }=\sigma _L/\sqrt{N}$ (see Table 1), where *N* is the number of simulated photons. Thus, in a verified MC code, the deviation between $\langle L \rangle _{MC}$ and $\langle L \rangle _{IP}$ can be made arbitrarily small by increasing the number of simulated trajectories. Most importantly, the knowledge of both $\langle L \rangle _{IP}$ and $\sigma _{\langle L \rangle }$, allows one to run one-sample t-tests to estimate the accuracy of a MC code.

This method is a powerful tool for detecting errors in a forward solver MC code, not only due to incorrect propagation models and/or in the implementation, but also due to lack of accuracy in the functions used, as well as in the random number generator (Table 3). The results here presented support such claims, showing both the power and the simplicity of the proposed method. The use of inhomogeneous media and refractive index mismatches emphasizes its intrinsic robustness for testing reflection and refraction phenomena at the boundary of two regions and for the calculation of partial pathlengths. Therefore, the method is particularly sensible to detect errors in treating the boundaries of the investigated medium and also those in the calculations of partial pathlengths in distinct regions of the medium. It is worth to note that the tight relation between fluence and mean pathlength for a Lambertian illumination, may be exploited in the future to propose a verification method based on the fluence^55^. The method can be applied to very simple geometries, but, as shown in the presented results, also to layered distributions of refractive index and scattering properties for which other exact analytical solutions of photons transport do not exist. Thus, the invariant solutions used offer a unique set of closed form solutions derived by the RTE that can cover a huge range of physical/geometrical situations and regimes of light propagation that cannot be found in other benchmarks used for radiative transfer problems.

Indeed, the independence of the exact solutions used from the scattering properties determines the main advantage of the method, i.e., its huge range of applicability from ballistic to diffusive regimes of propagation, also determines its main limitation that is its insensitiveness to the characteristic of scattering phase function. About the correct treatment of the scattering function inside the simulation there are other statistical tests that can be implemented as those that can be done with the benchmarks obtained by Zaccanti et al.^61^.

The simplicity of the used formulas makes the verification process straightforward, avoiding the use of reference values based on complex solution of the RTE (available only for a few media) or asymptotic behaviours obtained by solving the diffusion equation. It is also worth to note that in the case here reported the results are verified with up to six significant digits, that is with an accuracy higher than that of typical reference values used in biomedical optics^43,44^. Moreover, the numerical results were obtained by using an ordinary computer and therefore a larger number of verified significant digits can be achieved by this method with more powerful machines, implementing a larger number of simulated trajectories. In principle, such a number of digits is only limited by the computation time necessary to simulate the required number of trajectories and by the machine precision. This characteristic of the method is shared with other methods based on exact analytical solutions of the transport equation, while lower performances are expected when semi-analytical solutions are used.

Although the proposed method can only be applied to non-absorbing media, this fact does not represent an actual limitation of this procedure. Indeed, absorption is a physical effect that does not modify photons trajectories and it can be accounted for in a straightforward way according to the Beer–Lambert law of absorption. In other words, once a Monte Carlo code is verified for non-absorbing media, the inclusion of absorption does not involve critical computation issues inside the code. Thus, by adding an absorption coefficient $\mu _a$ inside a region *k* of the medium, the probability to detect each photon trajectory is modified by the scaling factor $\exp (-\mu _a \ell _k)$, with $\ell _k$ path length spent inside the region *k* by the trajectory. The correct application of this scaling relationship requires that the MC code returns the correct distribution for $\ell _k$. And this fact is guaranteed when, for the non-absorbing medium, $\langle \ell _k \rangle$ converges to the IP value and thus the verification method is successfully applied.

The proposed method exploits the invariance property of the mean path length that represents a reference standard in any scattering non-absorbing medium. Since the invariance of $\langle L \rangle$ derives from a more fundamental invariance property of the solutions for the radiance of the continuous wave RTE^55^ (under the hypothesis of Lambertian illumination), further development of this method for testing forward solver MC codes can involve exact invariant analytical solutions for radiance or fluence rate^55^. In fact, reference solutions for the radiance and other radiometric quantities such as the fluence rate are already available when a non-absorbing medium is illuminated with uniform isotropic light^55^. This aspect can be particularly important for the forward solver MC codes developed for complex geometries since the calculation of the fluence is a relevant parameter for many applications^34–40,42^.

In this work, we have used symmetries of the media (i.e., the geometry, and the distribution of the scattering properties and refractive indices) for speeding up the convergence of the MC simulations and for writing simpler codes. This allowed us to use Lambertian illumination at one point of the external boundary and to detect photons at all the points of the external boundary. The average pathlength calculated in this situation is the same as the average pathlength predicted by the IP. However, we stress that the applicability of this method is general and does not require any symmetry. An example of uniform Lambertian illumination on an inhomogeneous 2D medium (a circle) can be found in the of work of Tommasi et al.^52^ (see Figs. 2 and 7 of the paper) that shows the feasibility of the method with inhomogeneous and non symmetric media. Additionally, we note that a Lambertian illumination in a MC code, can be implemented also in complex geometries as it has been shown in recent literature^36^. Therefore, we envision that this method can be used, with some extra computational cost, as a general method for testing the correctness of a MC code directly for a situation of interest, no matter how complex it is, without resorting to simpler geometries and uniform distribution of the scattering properties. The only requirements is a uniform Lambertian illumination of its external boundary. With the current proliferation of accelerated MC codes based on graphic processor units (GPU), this type of illumination might be possible and the convergence of this method might be fast enough to encourage widespread use. Therefore, for a general medium the proposed method can be summarized in the following three points.

1. For any medium of interest, identified by its geometry, distribution of optical properties and refractive indices at its interior, we consider the same medium but with no absorption.
2. For this non-absorbing medium, we run a set of *N* trajectories upon the condition of uniform Lambertian illumination and calculate $\langle L \rangle _{MC}$ and its standard deviation $\sigma _{\langle L \rangle }$.
3. We apply a one sample t-test to determine whether $\langle L \rangle _{MC}$ is consistent with $\langle L \rangle _{IP}$.

As noted by Ganapol^30^ “It should be emphasized that there is no guaranteed method of verifying that a particular computational algorithm performs correctly for all cases envisioned. The value of the tests mentioned is in indicating algorithmic inconsistencies through their failure.” The method here presented does not make any exception to this fact: it works for detecting inconsistencies in a MC for transport problems, but it does not guarantee that the code will calculate correctly for all the envisioned cases and all the parameters of interest. It is actually a simple test, with a wide range of applicability, that informs of the presence of bugs inside a MC code. The bugs that can be detected with this verification method are primarily related to the treatment of boundaries and partial pathlengths inside tissue regions. However, it is worth to note that the exact type of bugs affecting the code cannot be precisely identified by the method that provides a warning of violation of the invariance property.

The applicability of the proposed method to any domain is also subjected to the requirement that volume and surface area are analytically calculable, otherwise the same accuracy of the results here described cannot be reproduced. Although the method is useful also if only applicable to some geometries, this issue needs some comments for the future developments. Monte Carlo codes can treat complex geometries using voxels or meshes. Complex geometries can thus be represented (voxelized) as superposition of voxels, and since each voxel is a known geometry where the invariance property can be applied as for the whole volume, this fact does not obstruct the application of the method. The closed form solutions used are applicable to any sub-volume of the medium: this fact extends the applicability of the method to domains expressed as a superposition of known voxels. In this work we have checked the basic application of the method: next step will be to verify its applicability to more complex geometries. However, the cases here considered, as for instance the one-hundred layered sphere, cannot be simply classified as exact RTE analytical solutions for elementary geometries since no semi-analytical solution exists for these cases.

The extension of the method to Monte Carlo developed in other fields than optics is straightforward whenever the validity of Eqs. (1)–(3), valid in optics, can be extended to the propagation of other random walkers. Whenever a random walker can be described by a transport equation, very similar solutions to those of Eqs. (1)–(3) can be obtained. It is thus exploited the extreme simplicity of the invariance solutions for the mean pathlength that holds for very different types of random walkers^49–51^.

It must be finally noted that, although here we have addressed only the case of elastic scattering, in principle the invariance property can also be extended to inelastic scattering or to branching random walks as neutron transport in a nuclear reactor^14,57^. This fact suggests that the applicability of the method may be extended to a larger range of forward solver MC codes.

In conclusion, the method proposed in this paper uses a unique reference standard that offers ideal conditions for the verification of Monte Carlo computational codes used in photon migration through turbid media. The method is particularly robust to test the effects of refractive index mismatch by handling the detailed information calculated with the mean path length. This fact implies that, given the tight relation between mean path length and fluence rate, also the information on the fluence rate can be in principle used and tested with the same level of accuracy. The method can be adapted in a straightforward way to the verification of computational codes for general transport processes. Thus, we believe that the method here proposed is an efficient procedure to generate reference numerical phantoms for any MC code that simulates random walks and offering the possibility to validate the code with arbitrary accuracy.

## Methods

### Illumination of the medium

The correct uniform isotropic illumination of the medium is usually carried out by exploiting the intrinsic symmetries of the considered geometries that allow to replace the uniform illumination on the external surface by a single point illumination. Thus, to retrieve a large number of data and to avoid problems in the sampling of a complicated medium surface, the intrinsic symmetries of some geometries, such as layered sphere, layered infinite slab and cylinder, has been used^62,63^. It is worth to note that this approach, apart the computational advantage, is mandatory only for the laterally infinitely extended slab geometry for which a uniform illumination cannot be practically implemented in a MC code due to the infinite dimension of the external boundary. For the slab it is also needed to switch alternatively the illumination of the two sides of the slab and also a double precision random number generator must be used. For the extraction of the entrance angle inside the slab we have used the rule given in “Special tests” section by using the probability density function of a Lambertian illumination. The Lambertian illumination is implemented by extracting the entrance angle with a cosine law distribution. We also note that the number of the simulated trajectories for the calculation of $\langle L \rangle$ coincides with the number of the received photons, i.e., such a number is not a random value.

### Step length distribution

An important requirement for an efficient implementation of the method is the use of an exponential probability density function for the length between two consecutive scatting events. Such a distribution is a consequence of the consideration that $\mu _s$ is a property of the medium in a certain point, independently by the rest of the volume. Such a characterization of the scattering medium is able to reproduce the propagation in many turbid media where each scattering event can be considered independent by the other ones. Moreover, the memoryless nature of the exponential distribution allows to take into account the boundary of the medium without introduction of any corrective term in the length step distribution. This is indeed the approach of the RTE in its standard formulation. Once the code is verified for the treatment of the trajectories, other kind of steps distributions can be inserted in the MC by using the correct boundary conditions.

### Verified Monte Carlo code

The MC core here subjected to verification is a program used for simulating the light propagation through turbid media in tissue optics. The core of the program, developed during the 1980s and 1990s Refs.^3,7,64–66^, is the generation of a large number of random trajectories, taking into account the modification in the scattering coefficient and in the refractive index inside the material. Before the present verification, the MC results were compared, with an excellent agreement, with exact solutions of the position where different orders of scattering occurs inside a non-absorbing infinite medium^3,61^.

Each length $\ell$ between two consecutive scattering events is drawn by an exponential probability density function:

4 $$\begin{aligned} p(\ell )=\mu _s\exp \left( -\mu _s\ell \right) . \end{aligned}$$

Then, each length is achieved by the inversion of the associated cumulative probability distribution function:

5 $$\begin{aligned} \ell (\xi )=-\frac{\ln \left( 1-\xi \right) }{\mu _s}, \end{aligned}$$

where $\xi$ is an uniformly distributed random number $\in (0,1)$. Monte Carlo simulations are often carried out implementing the equivalent expression $\ell (\xi )=-\frac{\ln \left( \xi \right) }{\mu _s}$. Also the intersection of the trajectory with the interface between two different media is considered as a scattering event in terms of new length extraction.

Concerning the scattering angles, the probability density is the Henyey-Greenstein, whose corresponding cumulative probability function is analytically invertible (see the analytical expression of the Henyey–Greenstein scattering function that can be found in literature^3,59^). Such a choice allows to avoid problems of sampling of the scattering angle distribution calculated with Mie theory.

## Supplementary Information

Supplementary Information.
