# Supplementary material for: Verification method of Monte Carlo codes for transport processes with arbitrary accuracy
Source: Sci Rep. 2021 Sep 30;11:19486. doi: 10.1038/s41598-021-98429-3 (PMC8484597; doi:10.1038/s41598-021-98429-3)
Supplement: Supplementary file 1 — Supplementary Information. [file 41598_2021_98429_MOESM1_ESM.pdf]

Verification Method of Monte Carlo codes for transport processes with arbitrary accuracy: Supplementary Material

Fabrizio Martelli[1], Federico Tommasi[1], Angelo Sassaroli[2], Lorenzo Fini[1] & Stefano Cavalieri[1]

[1]*Dipartimento di Fisica e Astronomia dell'Università degli Studi di Firenze, via Giovanni Sansone 1, 50019, Sesto Fiorentino, Italy*

[2]*Tufts University, Department of Biomedical Engineering, 4 Colby Street, Medford, Massachusetts 02155, USA*

In this document the tables of data in the case of the homogeneous sphere with different relative refractive index n_r and asymmetric factor g are shown, as well as figures pertain also different media (infinite cylinder and slab). The mean path length $\langle L \rangle$ are reported for different values of the scattering coefficient μ_s, for different asymmetric factor g of the scattering phase function and the number N of generated trajectories.

For very low scattering, we note a slower convergence of the MC results to the IP value when $n_r > 1$ compared to the other values of n_r. The reason of this behaviour lies in the presence of rare photons trajectories that when $n_r > 1$ can be established inside the volume with a regime of guided propagation. The guided propagation of this kind of trajectories can only be neutralized by one scattering event inside the medium. The effect is present for the geometries of sphere, slab and cylinder. The insufficient sampling of this kind of trajectories may lead to an inaccurate evaluation

$\langle L\rangle_{IP} = 26.\bar{6}$ mm	N=10^5		N=10^6		N=10^7		N=10^8		N=10^9	
μ_s (mm^{-1})	$\langle L\rangle$ (mm)	$\sigma_{\langle L\rangle}$ (mm)	$\langle L\rangle$ (mm)	$\sigma_{\langle L\rangle}$ (mm)	$\langle L\rangle$ (mm)	$\sigma_{\langle L\rangle}$ (mm)	$\langle L\rangle$ (mm)	$\sigma_{\langle L\rangle}$ (mm)	$\langle L\rangle$ (mm)	$\sigma_{\langle L\rangle}$ (mm)
10^{-5}	23.3	5.6	27.0	3.0	26.90	0.84	26.67	0.25	26.526	0.082
10^{-4}	23.0	2.3	26.15	0.74	26.82	0.22	26.544	0.075	26.639	0.023
10^{-3}	26.80	0.83	26.44	0.24	26.721	0.082	26.677	0.026	26.6686	0.0076
10^{-2}	26.18	0.23	26.752	0.081	26.672	0.029	26.6736	0.0093	26.6623	0.0029
10^{-1}	26.46	0.11	26.640	0.034	26.6714	0.0099	26.6640	0.0036	26.6659	0.0011
1	26.656	0.097	26.674	0.030	26.673	0.010	26.6690	0.0029	26.66728	0.00091

Table 1: Data for the sphere of radius $r = 5$ mm and $n_r = 2$ for different scattering coefficient μ_s and with HG scattering function $g = 0.9$. The value predicted by theory is $\langle L\rangle_{IP} = \frac{4}{3} r n_r^2 = 26.\bar{6}$ mm. The values for $\mu_s = 0$ are not reported because the IP is not valid in this case ($n_r > 1$ and no scattering).

of $\langle L\rangle$. The effect is particularly important for very low values of the scattering coefficient while it vanishes as the scattering increases.

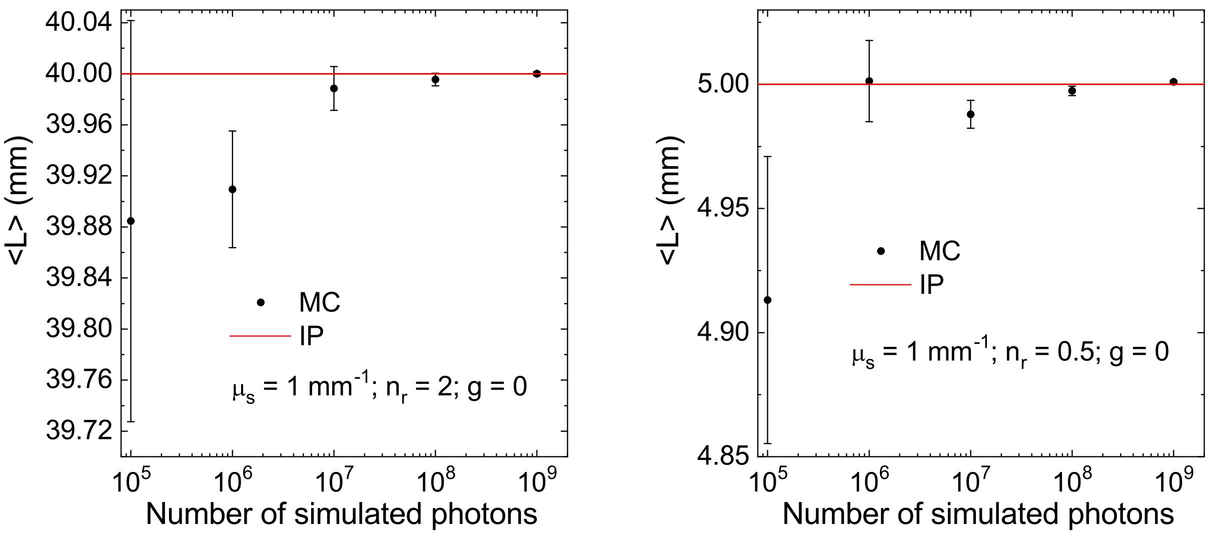

Figure 1: Left panel: $\langle L \rangle$ *for an homogeneous infinite cylinder of radius 5 mm, with* $n_r = 2$*. Right panel:* $\langle L \rangle$ *for an homogeneous infinitely extended slab of thickness 10 mm, with* $n_r = 0.5$*. For both figures the scattering coefficient and the asymmetry factor are respectively* $\mu_s = 1$ *mm*$^{-1}$ *and* $g = 0$*.*

$\langle L\rangle_{IP} = 26.\bar{6}$ mm	N=10^5		N=10^6		N=10^7		N=10^8		N=10^9	
μ_s (mm^{-1})	$\langle L\rangle$ (mm)	$\sigma_{\langle L\rangle}$ (mm)	$\langle L\rangle$ (mm)	$\sigma_{\langle L\rangle}$ (mm)	$\langle L\rangle$ (mm)	$\sigma_{\langle L\rangle}$ (mm)	$\langle L\rangle$ (mm)	$\sigma_{\langle L\rangle}$ (mm)	$\langle L\rangle$ (mm)	$\sigma_{\langle L\rangle}$ (mm)
10^{-5}	9.78	0.30	23.4	2.8	26.8	1.0	26.00	0.41	26.49	0.12
10^{-4}	27.8	3.8	26.5	1.0	26.88	0.38	26.56	0.15	26.6721	0.0356
10^{-3}	26.2	1.2	26.86	0.39	26.68	0.13	26.639	0.043	26.6539	0.0104
10^{-2}	26.09	0.39	26.53	0.13	26.670	0.040	26.670	0.014	26.6600	0.0038
10^{-1}	26.53	0.14	26.635	0.047	26.658	0.013	26.6648	0.0045	26.6666	0.0013
1	26.591	0.099	26.656	0.033	26.6784	0.0095	26.6730	0.0030	26.66705	0.00092

Table 2: Data for the sphere of radius $r = 5$ mm and $n_r = 2$ for different scattering coefficient μ_s and with HG scattering function $g = 0$. The value predicted by theory is $\langle L\rangle_{IP} = \frac{4}{3}r = 26.\bar{6}$ mm. The values for $\mu_s = 0$ are not reported because the IP is not valid in this case ($n_r > 1$ and no scattering).

$\langle L\rangle_{IP} = 1.\bar{6}$	N=10^5		N=10^6		N=10^7		N=10^8		N=10^9	
μ_s (mm^{-1})	$\langle L\rangle$ (mm)	$\sigma_{\langle L\rangle}$ (mm)	$\langle L\rangle$ (mm)	$\sigma_{\langle L\rangle}$ (mm)	$\langle L\rangle$ (mm)	$\sigma_{\langle L\rangle}$ (mm)	$\langle L\rangle$ (mm)	$\sigma_{\langle L\rangle}$ (mm)	$\langle L\rangle$ (mm)	$\sigma_{\langle L\rangle}$ (mm)
0	1.682	0.011	1.6608	0.0036	1.6685	0.0011	1.66594	0.00032	1.66670	0.00010
10^{-16}	1.688	0.012	1.6670	0.0032	1.6655	0.0010	1.66675	0.00034	1.66664	0.00010
10^{-11}	1.662	0.013	1.6706	0.0038	1.6651	0.0011	1.66659	0.00043	1.66669	0.00012
10^{-6}	1.661	0.012	1.6706	0.0041	1.6684	0.0009	1.66662	0.00036	1.66662	0.00011
10^{-5}	1.676	0.011	1.6637	0.0035	1.6675	0.0012	1.66710	0.00035	1.66678	0.00013
10^{-4}	1.665	0.013	1.6672	0.0039	1.6675	0.0011	1.66713	0.00038	1.66661	0.00010
10^{-3}	1.677	0.012	1.6622	0.0034	1.6657	0.0011	1.66658	0.00037	1.66659	0.00010
10^{-2}	1.674	0.011	1.6631	0.0042	1.6674	0.0011	1.66714	0.00039	1.66666	0.00011
10^{-1}	1.663	0.013	1.6659	0.0037	1.6653	0.0013	1.66707	0.00037	1.66663	0.00012
1	1.653	0.015	1.6729	0.0055	1.6656	0.0019	1.66641	0.00052	1.66667	0.00017

Table 3: Data for the sphere of radius $r = 5$ mm and $n_r = 0.5$ for different scattering coefficient μ_s and with HG scattering function $g = 0$. The value predicted by theory is $\langle L\rangle_{IP} = \frac{4}{3} r n_r^2 = 1.\bar{6}$ mm.

$\langle L\rangle_{IP} = 1.\bar{6}$	N=10^5		N=10^6		N=10^7		N=10^8		N=10^9	
μ_s (mm^{-1})	$\langle L\rangle$ (mm)	$\sigma_{\langle L\rangle}$ (mm)	$\langle L\rangle$ (mm)	$\sigma_{\langle L\rangle}$ (mm)	$\langle L\rangle$ (mm)	$\sigma_{\langle L\rangle}$ (mm)	$\langle L\rangle$ (mm)	$\sigma_{\langle L\rangle}$ (mm)	$\langle L\rangle$ (mm)	$\sigma_{\langle L\rangle}$ (mm)
10^{-4}	1.662	0.011	1.664	0.004	1.6675	0.0011	1.6663	0.0004	1.66647	0.00012
10^{-3}	1.654	0.013	1.663	0.003	1.6664	0.0011	1.6670	0.0004	1.66677	0.00011
10^{-2}	1.667	0.010	1.664	0.004	1.6689	0.0010	1.6666	0.0003	1.66654	0.00013
10^{-1}	1.647	0.011	1.662	0.003	1.6667	0.0011	1.6668	0.0004	1.66673	0.00011
1	1.653	0.012	1.670	0.004	1.6656	0.0012	1.6669	0.0004	1.66666	0.00013
10	1.661	0.018	1.677	0.005	1.6680	0.0018	1.6663	0.0006	1.66650	0.00018

Table 4: Data for the sphere of radius $r = 5$ mm and $n_r = 0.5$ for different scattering coefficient μ_s and with HG scattering function $g = 0.9$. The value predicted by theory is $\langle L\rangle_{IP} = \frac{4}{3} r n_r^2 = 1.\bar{6}$ mm.

$\langle L\rangle_{IP} = 6.\bar{6}$ mm	N=10^4		N=10^5		N=10^6		N=10^7		N=10^8		N=10^9		N=10^{10}	
μ_s (mm^{-1})	$\langle L\rangle$ (mm)	$\frac{\langle L\rangle-\langle L\rangle_{IP}}{\sigma_{\langle L\rangle}}$	$\langle L\rangle$ (mm)	$\frac{\langle L\rangle-\langle L\rangle_{IP}}{\sigma_{\langle L\rangle}}$	$\langle L\rangle$ (mm)	$\frac{\langle L\rangle-\langle L\rangle_{IP}}{\sigma_{\langle L\rangle}}$	$\langle L\rangle$ (mm)	$\frac{\langle L\rangle-\langle L\rangle_{IP}}{\sigma_{\langle L\rangle}}$	$\langle L\rangle$ (mm)	$\frac{\langle L\rangle-\langle L\rangle_{IP}}{\sigma_{\langle L\rangle}}$	$\langle L\rangle$ (mm)	$\frac{\langle L\rangle-\langle L\rangle_{IP}}{\sigma_{\langle L\rangle}}$	$\langle L\rangle$ (mm)	$\frac{\langle L\rangle-\langle L\rangle_{IP}}{\sigma_{\langle L\rangle}}$
0	6.645	-0.86	6.6760	1.38	6.6650	-0.73	6.66731	0.78	6.66650	-0.83	6.666658	-0.11	6.666698	1.12
10^{-7}	6.644	-0.91	6.6753	1.3	6.6672	0.22	6.66743	0.99	6.66683	0.68	6.666599	-0.86	6.666676	0.42
10^{-6}	6.616	-2.4	6.6667	0.0043	6.6628	-1.8	6.66606	-0.84	6.66683	0.65	6.666660	-0.095	6.666678	0.49
10^{-5}	6.636	-1.5	6.6624	-0.53	6.6690	1.1	6.66649	-0.23	6.66644	-0.94	6.666544	-1.7	6.666637	-1.4
10^{-4}	6.656	-0.44	6.6673	0.088	6.6622	-1.8	6.66607	-0.74	6.66672	0.23	6.666762	1.2	6.666677	0.37
10^{-3}	6.678	0.53	6.6727	0.74	6.6665	-0.069	6.66708	0.52	6.66666	-0.023	6.666606	-0.85	6.666687	0.78
10^{-2}	6.618	-1.9	6.6655	-0.16	6.6699	1.3	6.66744	1.0	6.66645	-1.0	6.666737	0.86	6.666669	0.097
10^{-1}	6.645	-0.64	6.668	0.12	6.6687	0.60	6.6668	0.13	6.66642	-0.70	6.66655	-1.1	6.666664	-0.081
1	6.714	0.54	6.703	1.3	6.6640	-0.33	6.6612	-2.3	6.66543	-1.3	6.66671	0.16	6.666559	-1.2
10	6.41	-0.68	6.63	-0.33	6.697	0.87	6.670	0.26	6.6673	0.15	6.6656	-0.89	6.66687	0.56

Table 5: Data for the sphere of radius $r = 5$ mm and $n_r = 1$ for different scattering coefficient μ_s and different numbers N of generated trajectories. The value predicted by theory is $\langle L\rangle_{IP} = \frac{4}{3} r = 6.\bar{6}$ mm. $\sigma_{\langle L\rangle}$ is the standard deviation of $\langle L\rangle$.

	$\langle L_1\rangle_{IP} = 2.490833$ mm		$\langle L_2\rangle_{IP} = 1.110000$ mm		$\langle L_3\rangle_{IP} = 0.395833$ mm		$\langle L_4\rangle_{IP} = 0.106667$ mm	
μ_{s0} (mm^{-1})	$\langle L_1\rangle$ (mm)	$\frac{\langle L_1\rangle-\langle L_1\rangle_{IP}}{\sigma_{\langle L_1\rangle}}$	$\langle L_2\rangle$ (mm)	$\frac{\langle L_2\rangle-\langle L_2\rangle_{IP}}{\sigma_{\langle L_2\rangle}}$	$\langle L_3\rangle$ (mm)	$\frac{\langle L_3\rangle-\langle L_3\rangle_{IP}}{\sigma_{\langle L_3\rangle}}$	$\langle L_4\rangle$ (mm)	$\frac{\langle L_4\rangle-\langle L_4\rangle_{IP}}{\sigma_{\langle L_4\rangle}}$
0	2.49068	-0.23	1.10999	-0.019	0.39576	-0.21	0.10699	1.7
10^{-6}	2.49039	-0.73	1.11017	0.32	0.39575	-0.25	0.10653	-0.00041
10^{-5}	2.49045	-0.74	1.10986	-0.27	0.39594	0.30	0.10673	0.33
10^{-4}	2.49136	0.78	1.10990	-0.19	0.39556	-0.85	0.10667	0.017
10^{-3}	2.49136	0.85	1.11052	1.0	0.39574	-0.28	0.10662	0.26
10^{-2}	2.49064	-0.35	1.11020	0.36	0.39567	-0.45	0.10687	1.4
10^{-1}	2.49165	1.0	1.10923	-1.5	0.39590	0.19	0.10687	1.2
1	2.4904	-0.39	1.10981	-0.20	0.39681	1.9	0.10682	0.57

Table 6: Data for a 4-layered non-absorbing sphere of external radius $r = 5$ mm and internal radii 4, 3 and 2 mm. In the first layer, $r \in (4, 5)$ mm, $n_1 = 1.75$ and $\mu_s = \mu_{s0}$; in the second layer, $r \in (3, 4)$ mm, $n_1 = 1.5$ and $\mu_s = 0$; in the third layer, $r \in (2, 3)$ mm, $n_1 = 1.25$ and $\mu_s = \mu_{s0}$; and in the forth layer $r \in (0, 2)$ mm, $n_1 = 1$ and $\mu_s = 0$. The refractive index of the external medium is 2. The results of MC simulations for several values of μ_{s0} are shown in table for the partial path length $\langle L_k\rangle$ spent in the layers. The values predicted by IP are also shown. The data in table pertain to $N = 10^7$ simulated trajectories.
